# Is transjugular insertion of a temporary pacemaker a safe and effective approach?

**DOI:** 10.1371/journal.pone.0233129

**Published:** 2020-05-12

**Authors:** Kwang Jin Chun, Hye Bin Gwag, Jin Kyung Hwang, Seung-Jung Park, Young Keun On, June Soo Kim, Kyoung-Min Park

**Affiliations:** 1 Division of Cardiology, Department of Internal Medicine, College of Medicine, Kangwon National University Hospital, Kangwon National University, Chuncheon, Korea; 2 Division of Cardiology, Department of Internal Medicine, Samsung Changwon Hospital, Sungkyunkwan University School of Medicine, Seoul, Korea; 3 Division of Cardiology, Department of Internal Medicine, Veterans Health Service Medical Center, Seoul, Korea; 4 Division of Cardiology, Department of Internal Medicine, Heart Vascular Stroke Institute, Samsung Medical Center, Sungkyunkwan University School of Medicine, Seoul, Korea; Baylor Scott and White, Texas A&M College of Medicine, UNITED STATES

## Abstract

Temporary pacemakers (TPMs) are usually inserted in an emergency situation. However, there are few reports available regarding which route of access is best or what the most preferred approach is currently in tertiary hospitals. This study aimed to compare procedure times, complication rates, and indications for temporary pacing between the transjugular and transfemoral approaches to TPM placement. We analyzed consecutive patients who underwent TPM placement. Indications; procedure times; and rates of complications including localized infection, any bleeding, and pacing wire repositioning rates were analyzed. A total of 732 patients (361 treated via the transjugular approach and 371 treated via the transfemoral approach) were included. Complete atrioventricular block was the most common cause of TPM placement in both groups, but sick sinus syndrome was especially common in the transjugular approach group. Separately, procedure time was significantly shorter in the transjugular approach group (9.0 ± 8.0 minutes vs. 11.9 ± 9.7 minutes; *P* < 0.001). Overall complication rates were not significantly different between the two groups, and longer duration of temporary pacing was a risk factor for repositioning. The risk of reposition was significantly increased when the temporary pacing was continued more than 5 days and 3 days in the transjugular approach group and the transfemoral approach group, respectively. The transjugular approach should be considered if the TPM is required for more than 3 days.

## Introduction

Temporary pacemakers (TPMs) are usually inserted in an emergency situation such as in the context of complete atrioventricular block or sinus node dysfunction with unstable vital signs. There are several routes of access for transvenous TPM placement; in fact, to date, all major venous access sites have been used one time or another for temporary pacing. However, each site had its own complications including discomfort, pneumothorax, lead instability, and infection. [1] One recent large-scale meta-analysis reported that the most preferred route of access is the right internal jugular vein followed by the subclavian and femoral veins. [2] This report also highlighted a complication rate and a difference between specialist and generalist outcomes, but most of the patients included in this study were treated before the 2000s and most of the TPMs were placed by generalists. [2] Even now, there are few reports available regarding which route of access is better or what the latest trend is in TPM placement among tertiary hospitals.

Given the above, in this study we aimed to compare indications of temporary pacing, procedure times, and complication rates between the transjugular and transfemoral TPM placement approaches performed by cardiologists. We also investigated risk factors for reposition of pacing wire and which route of access is better for temporary pacing.

## Materials and methods

### Study population and design

We retrospectively analyzed 810 consecutive patients who underwent TPM placement for any reason between April 2009 and March 2016. We only enrolled patients who were 20 years or older and who received a transjugular or transfemoral approach for TPM placement. Thus, we excluded patients aged 19 years or younger and those treated via other routes of access for temporary pacing.

Demographic data, indications, approaches, procedure times of TPM placement, complications, and repositioning rates were analyzed following a medical records review. We compared the parameters between patients treated with the transjugular approach or transfemoral approach, respectively.

The Institutional Review Board at Samsung Medical Center approved the study protocol (IRB No. 2016-04-149). The need for informed consent was waived for this retrospective study.

### Indications and approaches for transvenous temporary pacing

The indications for temporary pacing were classified into four categories: atrioventricular block, sick sinus syndrome, bradyarrhythmia related to acute myocardial infarction, and the need for transient temporary pacing during cardiac surgery or other procedures.

The venous access site of all patients who underwent TPM placement were analyzed using data from a medical records review, and only patients treated with the transjugular approach or transfemoral approach to TPM placement were enrolled in this study.

### Procedure times and complications of transvenous temporary pacing

The procedure time was defined as the time from initial venous puncture attempt to the achievement of appropriate positioning of the temporary pacing wire. We checked the procedure time using both a cardiac picture archiving and communication system (PACS) and chart review.

Pneumothorax, pericardial tamponade, cerebrovascular accident, and puncture site bleeding were classified as procedure-related complications. Any type of lead repositioning was classified as a non–procedure-related complication.

The duration of TPM use and complications, especially the pacing wire repositioning rate, were also evaluated.

### Statistics

Continuous variables are expressed as means ± standard deviations or medians and interquartile ranges. Categorical variables are expressed as frequencies and percentages. To compare the procedure time and duration of temporary pacing according to the route of access, we used the Student’s unpaired t-test for normally distributed data or the Mann–Whitney test for skewed data. Categorical variables were analyzed with the chi-squared test or Fisher’s exact tests. Binomial logistic regression analysis was used to calculate the hazard ratios (HRs) and 95% confidence intervals (CIs) for risk factors associated with the reposition of temporary pacing wire. Receiver operating characteristic (ROC) curve analysis was used to determine when reposition of a pacing wire was frequently performed. Calculations were performed using the Statistical Package for the Social Sciences version 20.0 for Windows software program (IBM Corp., Armonk, NY, USA). A *P*-value less than 0.05 was considered to be statistically significant.

## Results

**Fig 1:**
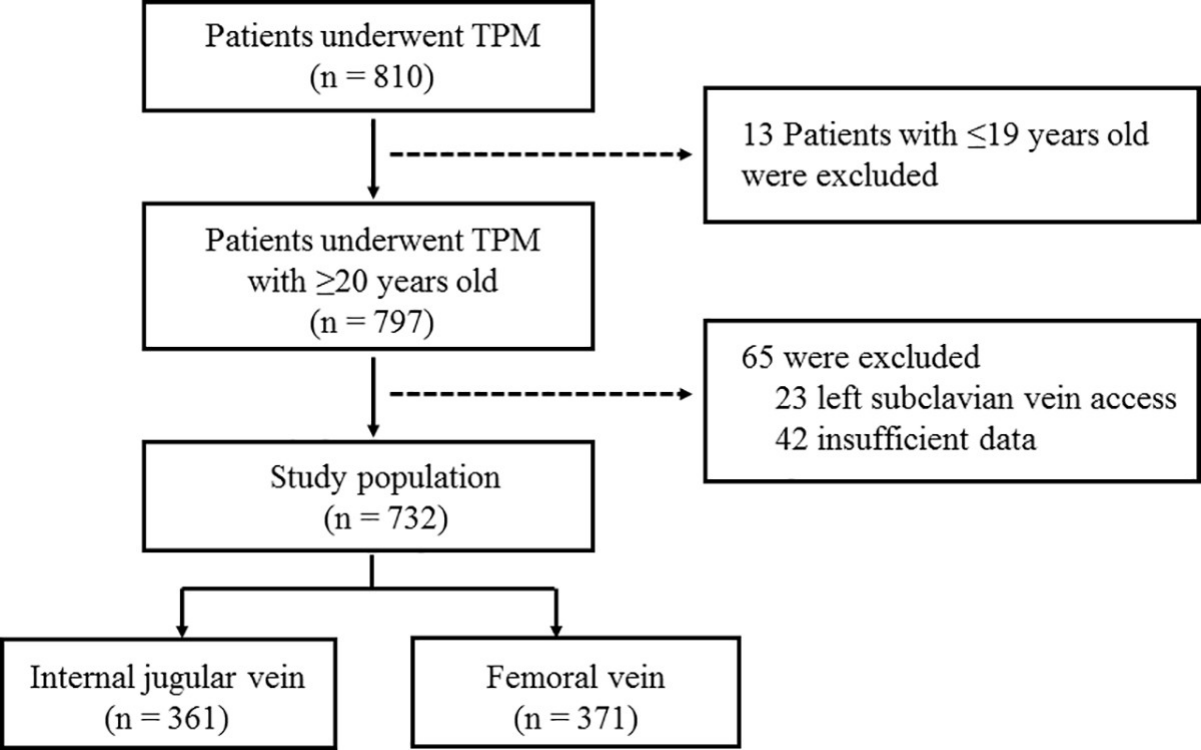
Enrollment of patients in the study.

Among the 810 patients who underwent TPM placement, 78 patients were excluded based on the following: 13 patients were younger than 20 years old, 23 patients received TPMs via the left subclavian vein, and 42 patients had insufficient data. Ultimately, a total of 732 patients were analyzed in this study (Fig 1).

### Baseline clinical characteristics of the study population

The mean age of the total study population was 67.1 ± 13.5 years, and 403 patients (55.1%) were male (Table 1). The mean age, percentage of males, body weight, and body mass index findings were not significantly different between the study groups. Hypertension, dyslipidemia, and a history of atrial fibrillation were more common in the transjugular approach group than the transfemoral approach group.

**Table 1:** Baseline characteristics of the study population.

	Transjugular approach group (n = 361)	Transfemoral approach group (n = 371)	*P*-value
Age (years)	67.4 ± 13.7	66.8 ± 13.3	0.407
Male sex (n, %)	194 (53.7)	209 (56.3)	0.480
Weight (kg)	61.1 ± 15.8	62.4 ± 13.5	0.120
BMI (kg/m^2^)	23.6 ± 4.3	23.7 ± 3.6	0.287
DM (n, %)	102 (28.3)	114 (30.7)	0.463
HTN (n, %)	190 (52.6)	235 (63.3)	0.003
Dyslipidemia (n, %)	17 (4.7)	32 (8.6)	0.034
AF (n, %)	100 (27.7)	51 (13.7)	< 0.001
CHF (n, %)	2 (0.6)	4 (1.1)	0.432
Stroke (n, %)	22 (6.1)	22 (5.9)	0.926

BMI, body mass index; DM, diabetes mellitus; HTN, hypertension; AF, atrial fibrillation; CHF, congestive heart failure

### Indication and procedure time according to TPM access type

Table 2 shows the indications and procedure times for TPM placement. Atrioventricular block was the most common cause of TPM use in both groups (n = 371; 51%), but the indication profiles were significantly different between the two groups (*P* = 0.003). Sick sinus syndrome was more common in the transjugular approach group than in the transfemoral approach group, while acute myocardial infarction was more common in the transfemoral approach group than in the transjugular approach group.

**Table 2:** Indications and procedure times according to TPM procedure access type.

	Transjugular approach group (n = 361)	Transfemoral approach group (n = 371)	*P*-value
Indication (n, %)			0.003
Atrioventricular block	177 (49.0)	194 (52.3)	
Sick sinus syndrome	170 (47.1)	117 (31.5)	
Acute myocardial infarction	8 (2.2)	39 (10.5)	
During other procedure	6 (1.7)	21 (5.7)	
Procedure time (minutes)	9.0 ± 8.0	11.9 ± 9.7	< 0.001
Duration of temporary pacing (days)	5 (3–7)	1 (1–3)	< 0.001

The procedure time for TPM placement was significantly shorter in the transjugular approach group (9.0 ± 8.0 minutes vs. 11.9 ± 9.7 minutes; *P* < 0.001). The median duration of temporary pacing was four days in the transjugular approach group, which was longer than that in the transfemoral approach group [5 days (IQR: 3–7 days) vs. 0 days (IQR: 1–3 days)]; *P* < 0.001].

### Overall complication rate and types of complications

The overall complication rate in this study across the total population was 7.4%. The complication rate was numerically higher in the transjugular approach group but not statistically significantly (8.9% vs. 5.9%; *P* = 0.129) (Table 3) when stratified by group. There were no episodes of procedure-related complications other than puncture site bleeding. Bleeding complications, including minor bleeding, were higher in the transfemoral approach group. Non–procedure-related complications including lead repositioning were higher in the transjugular approach group.

**Table 3:** Complication rates and types of complication.

	Transjugular approach group (n = 361)	Transfemoral approach group (n = 371)	*P*-value
Complication (n, %)	32 (8.9)	22 (5.9)	0.129
Type of complication			
Reposition (n, %)	28 (7.8)	11 (3.0)	
Pain (n, %)	2 (0.6)	0 (0.0)	
Bleeding (n, %)	2 (0.6)	11 (3.0)	

### Repositioning rate and causes of repositioning

Twenty-eight patients and 11 patients underwent repositioning of a temporary pacing wire during temporary pacing in the transjugular approach group and transfemoral approach group, respectively (7.8% vs. 3.0%; *P* = 0.004) (Table 4). However, this interpretation requires attention because the duration of temporary pacing was significantly longer in the transjugular approach group. The most common cause of repositioning was capture failure in both groups.

**Table 4:** Pacing wire repositioning rate and causes of repositioning.

	Transjugular approach group (n = 361)	Transfemoral approach group (n = 371)	*P*-value
Repositioning (n, %)	28 (7.8)	11 (3.0)	0.004
Cause of repositioning			
Capture failure (n, %)	25 (6.9)	6 (1.6)	
Infection (n, %)	1 (0.3)	1 (0.3)	
Discomfort (n, %)	0 (0.0)	4 (1.1)	
PNS (n, %)	1 (0.3)	0 (0.0)	
Bleeding (n, %)	0 (0.0)	0 (0.0)	

PNS, phrenic nerve stimulation

### Risk factors for repositioning

Univariate analysis showed that older age (*P* = 0.045), female sex (*P* = 0.005), body weight (*P* = 0.030), internal jugular vein as a route of access (*P* = 0.004), sick sinus syndrome as an indication for TPM placement (*P* = 0.024), and duration of temporary pacing (*P* <0.001) were associated with complications (Table 5). Binomial logistic regression analysis showed that female sex (HR: 2.532, 95% CI: 1.254–5.114; *P* = 0.010) and duration of temporary pacing (HR: 1.094, 95% CI: 1.032–1.159; *P* = 0.002) were risk factors for need for repositioning. Internal jugular vein access was not associated with repositioning in multivariate analysis.

**Table 5:** Univariate and multivariate analyses of need for repositioning.

Variable	Univariate analysis			Multivariate analysis	
	Repositioning (n = 39)	No repositioning (n = 693)	*P*-value	HR (95% CI)	*P*-value
Age	70.4 ± 14.0	66.9 ± 13.5-	0.045	1.017 (0.991–1.044)	0.211
Female sex	26 (66.7)	303 (43.7)	0.005	2.532 (1.254–5.114)	0.010
Weight	55.6 ± 13.9	62.1 ± 14.7	0.030	-	-
BMI	23.1 ± 3.9	23.7 ± 4.0	0.406	-	-
Internal jugular vein access	28 (71.8)	333 (48.1)	0.004	0.550 (0.259–1.167)	0.119
Sick sinus syndrome	22 (56.4)	265 (38.2)	0.024	1.799 (0.919–3.522)	0.086
Duration of TPM	7 (5–8)	3 (1–5)	< 0.001	1.094 (1.032–1.159)	0.002
Procedure time	9.4 ± 10.7	10.6 ± 9.0	0.227		

BMI, body mass index; HR, hazard ratio; CI, confidence interval; TPM, temporary pacemaker

### ROC analysis of duration of temporary pacing according to need for repositioning

**Fig 2:**
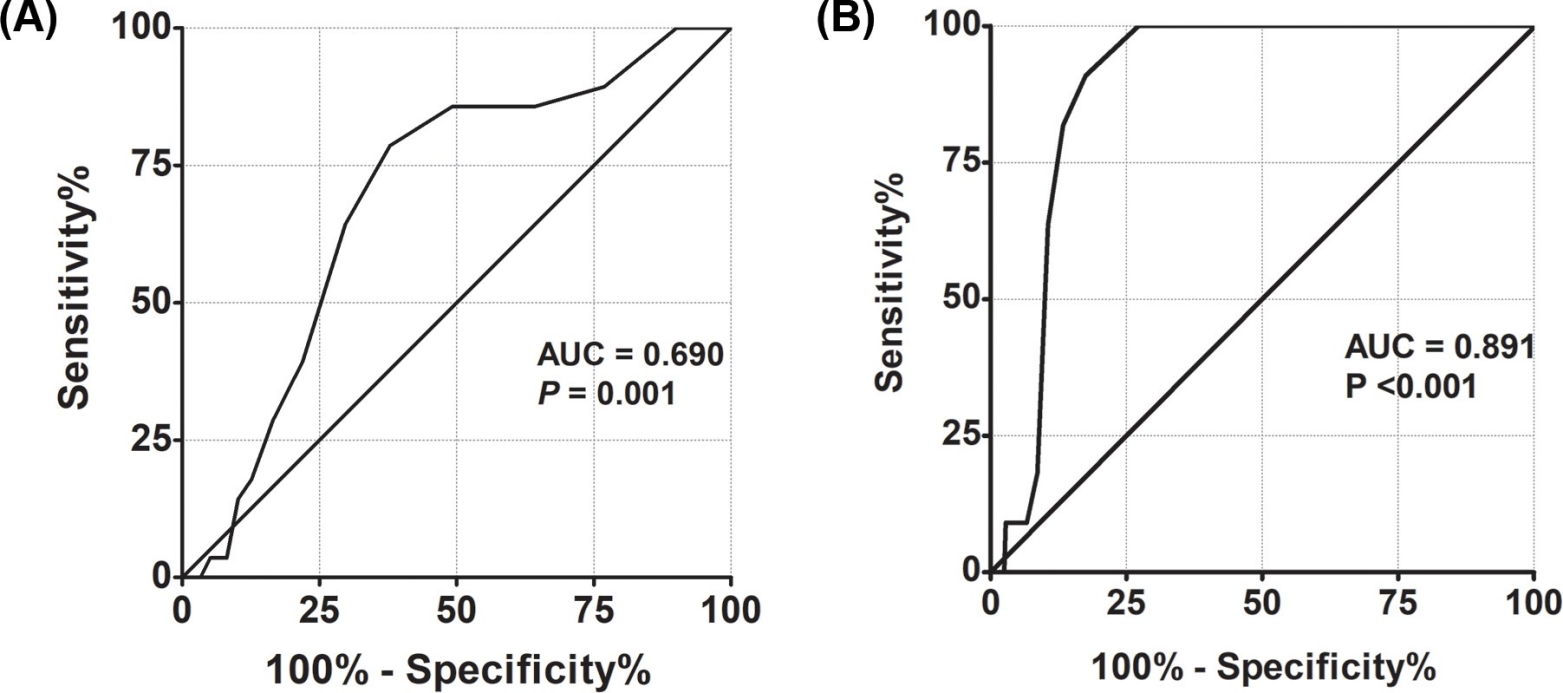
ROC curve of duration of temporary pacing to predict need for repositioning. ROC curve analysis of the transjugular approach group (A) and the transfemoral approach group (B). AUC = area under the curve, ROC = Receiver operating characteristic.

We performed ROC curve analysis to determine when reposition of pacing wire was frequently performed. ROC analysis for duration of temporary pacing as a risk factor of repositioning revealed an area under the curve of 0.690 (*P* = 0.001) and 0.891 (*P* <0.001) in transjugular approach group and transfemoral approach group, respectively (Fig 2). More than 5 days of temporary pacing through the internal jugular vein resulted in a sensitivity and specificity for need for repositioning of 78.6% and 62.2%, respectively. More than 3 days of temporary pacing through the femoral vein resulted in a sensitivity and specificity for need for repositioning of 90.9% and 82.5%, respectively.

## Discussion

The main findings of this study included that the most common cause of TPM is atrioventricular block and the procedure time is shorter among patients undergoing TPM placement using the transjugular approach as compared with the transfemoral approach. Separately, the overall incidence of complications is similar between the transjugular approach group and the transfemoral approach group, and longer duration of temporary pacing is a risk factor for need for repositioning.

TPMs are inserted in various situations and can be placed using various routes of access. [1] There exist arguments both in favor of and against all the major venous access sites (i.e., internal and external jugular, subclavian, brachial, or femoral). Hynes et al. [3] previously reported their five-year experience with TPM procedures in the coronary care unit. In their study, the most preferred route for access was antecubital vein cutdown (59.3%) followed by subclavian vein (17.3%) and right internal jugular vein (10.9%) use, but, ultimately, the right internal jugular approach was associated with the lowest complication rate in this study. [3] In other studies conducted during the similar period before 2000, the most common venous access site was the subclavian vein [4–6] or femoral vein. [7, 8] The British Cardiac Society recommends that the right internal jugular vein is the best route to use in most patients, particularly during procedures conducted by inexperienced operators, because this provides the most direct route to the right ventricle, a high success rate, and few complications. [9] Since this recommendation, other studies conducted after 2000 have reported that temporary pacing via the internal jugular vein is the most commonly used route. [10–12] In our study, the use of the internal jugular vein and the femoral vein as route of access was similar, but the indications for temporary pacing according to the access site used were significantly different. This difference in indication can be linked also to the duration of temporary pacing—that is, the duration of temporary pacing was longer in the transjugular approach group, which was mainly driven by the proportion of sick sinus syndrome, which required a significantly longer duration of temporary pacing than the other indications (*P* < 0.001). Since the use of the femoral route restricts the patient’s mobility, it is thought that femoral access is used more often when a short period of temporary pacing is required, such as in the context of bradyarrhythmia due to acute myocardial infarction, during another invasive procedure, or immediately before the implantation of a permanent pacemaker.

Complications are not uncommon and are reported to be in the range of 10% to 59.9%. [2] Earlier, the most common complication following the insertion of the temporary pacing wire was said to be the malfunction of the TPM. [2, 10, 11] In this study, the most common type of complication was also pacemaker malfunction, which necessitated repositioning of the pacing wire. The overall complication rate of our study was 7.4%, which is quite low when compared with other rates in previous studies. As the complication rate was low when the TPM was inserted by an experienced operator, [10] the reason for the low complication rate seen in our study could be that all involved operators were experienced cardiologists. Most previous studies discussed capture failure, which is the most common complication of TPM, as the result of a device malfunction or lead displacement, but the pacing wire repositioning rate has not been as widely reported. In our study, intermittent capture failure not leading to repositioning of the pacing wire was not regarded as a complication. This is a strength of our study, in that it has revealed the “real” repositioning rate. The higher repositioning rate in the transjugular approach group is believed to have been caused by the longer duration of TPM use in this group; in practice, the duration of TPM use in patients who underwent repositioning of the pacing wire was significantly longer than among those who did not (*P* < 0.001). Bleeding complications developed mostly in patients treated via the transfemoral approach and was probably caused by multiple attempts of femoral vein puncture or femoral artery puncture. The location of internal jugular vein in a position that is easily visible and compressible to medical staff is another reason for the lower risk of bleeding than when using the femoral vein access. Although the bleeding complication rate is low, the transjugular approach seemed to be safer than the transfemoral approach in regard to bleeding risk.

Our study showed that female sex and duration of temporary pacing were risk factors for repositioning. Using the internal jugular vein as an access route was not associated with repositioning of temporary pacing wire after adjusting for duration of temporary pacing. The risk of reposition was significantly increased when the temporary pacing was continued more than 5 days and 3 days in the jugular vein approach group and the femoral vein approach group, respectively. Therefore, if a temporary pacemaker is needed for more than 3 days, it would be better to use the transjugular approach rather than the transfemoral approach. It was not clear how female sex contributes to the repositioning of temporary pacing wire. Obesity was not a factor that could explain why female sex is a risk factor for repositioning. Further research in this regard is potentially needed.

This study had several limitations. First, this study was a non-randomized and single-center retrospective observational study. Even though medical chart and cardiac PACS reviews were carefully performed, we could not fully control for all variable parameters nor confirm the exact procedure time in some cases. Second, the definition of “complication” was arbitrary. Minor bleeding was included under the umbrella of bleeding complications in our study, but intermittent capture failure was not included as a complication. Moreover, because our study is based on data gleaned in part from a medical chart review, the results could be affected if any minor (or major) complications were not recorded. Third, we did not check the use of antibiotics before and after TPM placement. There is a lack of data on whether the use of antibiotics around the time of surgery helps to prevent localized infection or sepsis. Our study has not solved this problem either, so further research is warranted.

## Conclusion

The internal jugular vein and femoral vein were similarly used as routes to insert TPMs, and the most common indication for temporary pacing was atrioventricular block. The procedure time was significantly shorter in the transjugular approach group, but the overall incidence of complications was similar between the two groups. The duration of temporary pacing was a risk factor for repositioning need, and the transjugular approach should be considered if the TPM is required for more than 3 days.

## Supporting information

S1 Dataset Minimal relevant dataset of this study. (XLSX)
